# Comparative assessment of respiratory, hematological and inflammatory profiles of long-term users of cigarettes, shisha, and e-cigarettes in Saudi Arabia

**DOI:** 10.18332/tid/202350

**Published:** 2025-05-10

**Authors:** Mazen M. Homoud, Rowaida Qoutah, Gokul Krishna, Noran Harbli, Layan Saaty, Afrah Obaidan, Abdulrahman Alkhathami, Noran Jamil, Tala M. Alkayyat, Maryam Alsughayyir, Nada Gubari, Saleh Alkhathami, Ali Alqarni, Omar Alqurashi, Khalid Assiri, Khalid Saeed Alwadeai, Wafaa Abdulrahman, Husam Alahmadi, Ayedh Alahmari

**Affiliations:** 1 Department of Respiratory Therapy, Faculty of Medical Rehabilitation Sciences, King Abdulaziz University, Jeddah, Saudi Arabia; 2 Department of Respiratory Therapy, Batterjee Medical College, Jeddah, Saudi Arabia; 3 Department of Medical Laboratory, King Fahad Armed Hospital, Jeddah, Saudi Arabia; 4 Department of Rehabilitation Sciences, College of Applied Medical Sciences, King Saud University, Riyadh, Saudi Arabia; 5 Department of Microbiology, Batterjee Medical College, Jeddah, Saudi Arabia

**Keywords:** cigarettes, shisha, e-cigarettes, hematology, PFT

## Abstract

**INTRODUCTION:**

Globally, over 1 billion people smoke, resulting in approximately 8 million deaths each year. Although the health risks associated with traditional cigarettes are extensively documented, there is an increasing need to evaluate the long-term effects of alternative tobacco products, particularly shisha, and e-cigarettes. This study seeks to compare the respiratory, hematological, and inflammatory profiles of long-term users of cigarettes, shisha, and e-cigarettes in Saudi Arabia.

**METHODS:**

A cross-sectional, observational study was conducted at the Respiratory Therapy laboratories of Batterjee Medical College (BMC), Jeddah, Saudi Arabia, between February 2022 and August 2023. It involved four groups: cigarette smokers, shisha smokers, e-cigarette users, and non-smokers. Pulmonary function tests (PFTs) measured FEV_1_, FVC, and other lung function parameters. Hematological profiles, including WBC, neutrophils, lymphocytes, monocytes, and C-reactive protein (CRP) levels, were assessed.

**RESULTS:**

Cigarette and shisha users demonstrated significantly reduced FEV_1_ (cigarettes: 3.11 ± 0.54 L/s, shisha: 3.26 ± 0.71 L/s; p≤0.0001), FEV_1_ (% predicted: 81.63 ± 12.11 for cigarettes, 88.09 ± 12.92 for shisha; p≤0.0001), and FVC (3.87 ± 0.68 L for cigarettes, 3.95 ± 0.880 L for shisha; p=0.004), compared to non-smokers and e-cigarette users. Cigarette smokers exhibited significantly elevated WBC (7.92 ± 2.84 ×10^9^/L; p≤0.001), neutrophil (4.03 ± 2.29 ×10^9^/L), lymphocyte (2.95 ± 0.95 ×10^9^/L), and monocyte counts (0.67 ± 0.21 ×10^9^/L) compared to non-smokers, shisha, and e-cigarette users (p≤0.0001). Cigarette smokers and shisha smokers presented different lung function results but similar inflammatory profiles. However, e-cigarette users demonstrated comparatively higher lung function and lower inflammatory markers compared to both cigarette and shisha users.

**CONCLUSIONS:**

Compared to non-smokers, long-term cigarette and shisha smoking is associated with airway obstructive changes and increased inflammatory responses. Although e-cigarette users demonstrated lower inflammatory markers and less deviation from normal PFT, some exhibited borderline values with airway obstruction. Further research is needed to clarify the long-term health consequences of e-cigarette use.

## INTRODUCTION

Cigarette smoking remains the leading global risk factor for loss of disability-adjusted life years^1^, with over 1 billion smokers worldwide and the majority residing in low- and middle-income countries^2^. While smoking prevalence among the youth in Saudi Arabia has seen a decline, current rates remain a cause for concern. According to the latest Global Youth Tobacco Survey updated 7 September 2023, the proportion of students using any form of tobacco (including shisha and heated tobacco products) has decreased from 15.9% in 2007 to 9.4%. Cigarettes and shisha each account for 2.9% of youth smoking, with other tobacco products at 5.7%. Additionally, 1.5% of youth use heated tobacco, 3.6% use smokeless tobacco, and e-cigarette use has surged to 5.4%^3^. These figures highlight shifting patterns of tobacco consumption, particularly the rising popularity of e-cigarettes among younger populations.

Cigarette smoking remains the most prevalent form of tobacco use, exposing users to thousands of harmful chemicals, including nicotine, benzo(a)pyrene, carbon monoxide, acetaldehyde, hydrogen cyanide, benzene, formaldehyde, and heavy metals like cadmium and lead^4^. These substances contribute to various diseases by causing systemic absorption of toxins and localized pulmonary damage through oxidants and other harmful chemicals^4^. The toxic cocktail in cigarette smoke, including free radicals and fine particulates, plays a significant role in respiratory, cardiovascular, and other chronic diseases^5,6^.

Shisha, also known as hookah or waterpipe, involves inhaling flavored tobacco smoke through a water-filled pipe^7^. Despite the water filtration, shisha smoke contains high levels of toxic substances, including nicotine, tar, and heavy metals. The misconception that water filtration reduces harmful effects leads to its widespread use, particularly in social settings^8^.

E-cigarettes and vaping devices vaporize a liquid (usually containing nicotine, propylene glycol, vegetable glycerin, and flavorings) into an aerosol that users inhale^9^. Although marketed as a safer alternative to traditional smoking, e-cigarettes still pose health risks, including potential lung injury, nicotine addiction, and exposure to harmful substances such as formaldehyde and acrolein^10^. Their role in smoking cessation has been controversial, but a recent Cochrane review indicates evidence that e-cigarettes containing nicotine enhance quit rates compared to nicotine replacement therapies^11^. Additionally, moderate evidence supports the effectiveness of nicotine e-cigarettes in increasing quit rates relative to e-cigarettes without nicotine^12^. Cigars and pipes involve smoking tobacco in larger quantities than cigarettes, exposing users to toxic chemicals that are associated with increased risk of cancer in the head and neck, lungs, and liver, and increases the mortality rate^13,14^. Smokeless tobacco products, including chewing tobacco, snuff, and snus, are placed in the mouth or nose, where nicotine is absorbed through mucous membranes. While they bypass inhalation, smokeless tobacco still poses risks, such as oral cancers, gum disease, and nicotine addiction^15^.

Smoking remains a leading cause of death worldwide, contributing to cancers, cardiovascular diseases, and pulmonary disorders^16-20^. Annually, approximately 8 million deaths are attributed to smoking, including 1.3 million from secondhand smoke^9^. The economic burden from smoking is substantial, stemming from increased healthcare costs and lost productivity. While the health risks of traditional cigarettes are well documented, there is a growing need to assess the long-term impact of other forms of tobacco use, particularly shisha and e-cigarettes. This study aims to compare the respiratory, hematological, and inflammatory profiles among long-term users of cigarettes, shisha, and e-cigarettes in Saudi Arabia.

## METHODS

### Study design and setting

This cross-sectional, observational study was conducted at the Batterjee Medical College (BMC), Saudi Arabia, between February 2022 and August 2023. Participants were recruited using a convenience sampling method through social media advertisements and flyers distributed within the college. This study received ethical approval from the Institutional Review Board of Batterjee Medical College (RES 2022-43) in January 2022. All participants were informed about the purpose of the study and provided signed consent. Eligibility was determined through a comprehensive review of smoking history and predefined exclusion criteria to ensure the selection of suitable participants. Participants were assured of their right to withdraw at any time without consequences, and all data were de-identified by assigning unique codes and stored on a password-protected flash drive that was only accessible by the research team. Moreover, all procedures followed BMC IRB guidelines.

### Study protocol

**Figure 1:**
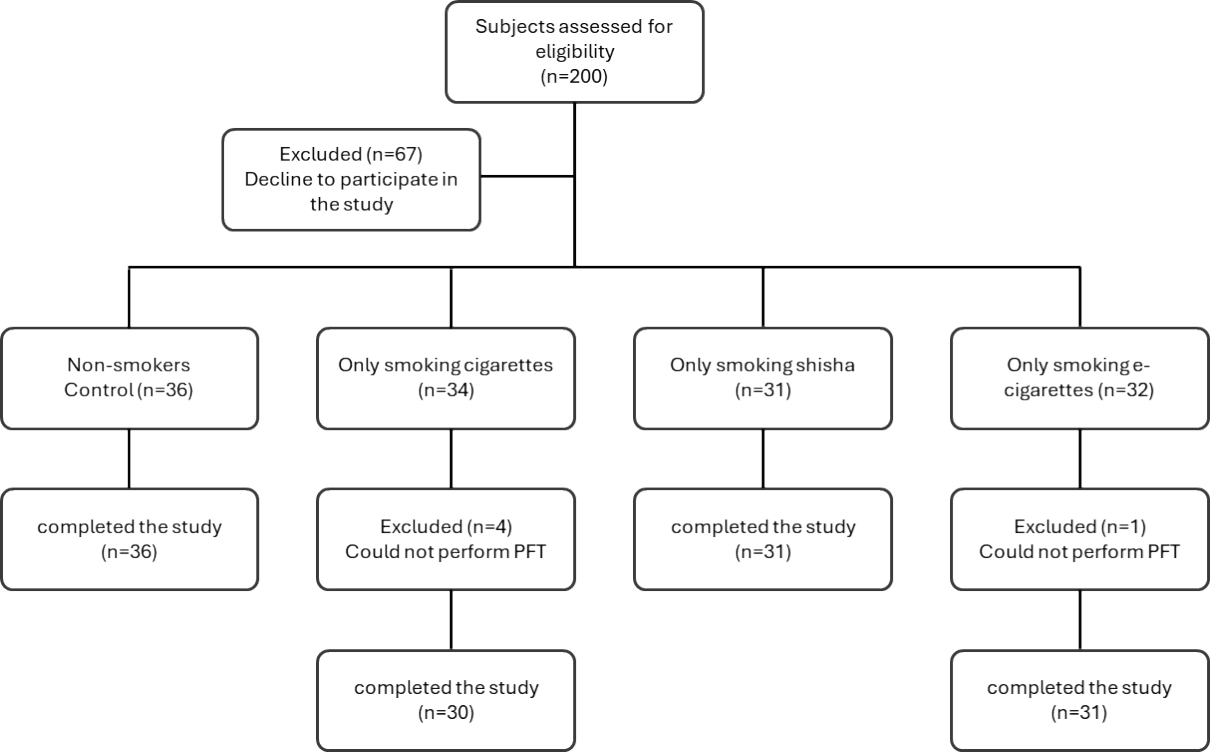
CONSORT flow chart illustrating participant selection from screening to inclusion in a cross-sectional observational study conducted in Saudi Arabia 2023 (N=128)

A total of 200 subjects were screened for eligibility, with 67 excluded for not meeting the inclusion criteria or declining participation. The remaining 133 participants were categorized into four groups based on their smoking habits: 1) Non-smoker group: participants who had never smoked or vaped; 2) Cigarette users: individuals with a history of daily cigarette smoking for at least four pack-years; 3) Shisha users: individuals who had smoked shisha daily for at least 4 years; and 4) E-cigarette users: participants with a history of daily e-cigarette use for at least 4 years. All participants were exclusive users of their respective smoking products, with no dual use. However, four participants from the cigarette group and one from the e-cigarette group were excluded due to an inability to perform the pulmonary function test (PFT) correctly, resulting in a final sample of 128 participants: 36 non-smokers, 30 cigarette users, 31 shisha users, and 31 e-cigarette users (Figure 1). All participants were aged ≥18 years and provided informed consent before joining the study.

Exclusion criteria included a history of unstable cardiopulmonary status, neuromuscular disorders, chronic lung disease, lung infections within the past 1–3 months, or the use of medications that could affect the complete blood count (CBC), PFT, or C-reactive protein (CRP), such as anti-inflammatories, antibiotics, and antihistamines. Individuals who identified as dual users (both traditional and electronic cigarettes) or those unable to perform the PFT test correctly were also excluded. All tests were conducted in the same building with a 30-minute break between each. A physician from the BMC clinic was available during the study in case of emergencies.

### Data variables

*Demographic data and medical history*

Information on age, gender, and smoking history was collected, along with medical history, to account for any pre-existing conditions that might influence the outcomes.

*Pulmonary function test (PFT)*

PFT was conducted using standardized protocols to measure forced expiratory volume in 1 s (FEV_1_), Forced vital capacity (FVC), and the FEV_1_/FVC ratio. Absolute values and percentages of predicted normal values (% predicted) were reported, following guidelines from the European Respiratory Society (ERS) and the American Thoracic Society (ATS)^21^. The tests were performed using the Master Screen PFT system powered by SentrySuite™ software (Vyaire Medical).

*Complete blood count (CBC) and C-reactive protein (CRP)*

Hematological parameters, including red blood cell count (RBC), hemoglobin (HGB), hematocrit (HCT), platelet count, white blood cell count (WBC), and differential leukocyte counts (eosinophils, neutrophils, lymphocytes, basophils, and monocytes) were measured via CBC. CRP levels were assessed to evaluate systemic inflammation. Venous blood samples were collected by trained phlebotomists following international guidelines from the Clinical and Laboratory Standards Institute (CLSI)^22^. CBC samples were stored in K3EDTA tubes, while CRP samples were collected in serum tubes. The Abbott Alinity H-Series system was used for CBC analysis, and the Abbott Alinity CI-Series system for CRP serum analysis. Blood samples were obtained within one week post-PFT to ensure data relevance.

### Sample size

*A priori* power analysis was conducted using G*Power version 3.1.9.7 to determine the appropriate sample size. To achieve 80% statistical power for detecting differences in the primary outcome variable (e.g. pulmonary function measures) among four groups. With a significance level of α=0.05 and a medium effect size (0.25), the analysis indicated that a minimum of 180 participants was required to achieve 80% statistical power using one-way ANOVA.

### Statistical analysis

The normality of data distribution was assessed using the Shapiro-Wilk test. Data conforming to normal distribution are presented as mean ± standard deviation (SD), while non-normally distributed data are expressed as median with interquartile range. Categorical variables are reported as frequencies with percentages. One-way ANOVA and Kruskal-Wallis tests were used to compare continuous variables across groups, with *post hoc* Tukey’s HSD test or Dunn’s pairwise test with Bonferroni correction applied to significant ANOVA results to determine inter-group differences. Statistical significance was set at p≤0.05, and analyses were performed using SPSS version 25.0 for Windows (Chicago, IL, USA).

## RESULTS

Gender distribution revealed a male predominance in all groups. The mean age of participants was 27.38 ± 5.75 years. Body mass index (BMI, kg/m^2^) differed significantly across the groups (p=0.012), with shisha users having the highest BMI. Table 1 summarizes the demographic characteristics of the participants.

**Table 1:** Sociodemographic characteristics of participants, a cross-sectional observational study conducted in Saudi Arabia, 2023 (N=128)

*Characteristics*	*Non-smokers* *(N=36)* *Median (IQR)*	*Cigarettes* *(N=30)* *Median (IQR)*	*Shisha* *(N=31)* *Median (IQR)*	*E-cigarettes* *(N=31)* *Median (IQR)*	*p*
**Female**, n (%)	3 (8)	4 (13)	10 (32)	4 (13)	0.087
**Age** (years)	25 (6)	28 (10.0)	28 (8.0)	23 (7)	0.012
**BMI** (kg/m^2^)	22.3 (4.67)	25.48 (7.34)	23.94 (6.66)	25.33 (7.16)	0.012
**Smoking duration** (years)	-	7.0 (7.5)	4.5 (6.8)	4.0 (2.5)	≤0.0001

BMI: body mass index.

### Lung function across smoking groups

Cigarette and shisha users exhibited significantly reduced FEV_1_ compared to non-smokers and e-cigarette groups. A one-way ANOVA demonstrated significant differences in FEV_1_ between groups (p≤0.0001). Tukey’s HSD *post hoc* analysis revealed significant mean differences between non-smokers and cigarette smokers (mean=0.61; 95% CI : 0.22–1.04; p=0.001), non-smokers and shisha smokers (mean= 0.46; 95% CI: 0.09–0.88; p=0.013), e-cigarette and cigarette smokers (mean= 0.68; 95% CI: 0.26–1.01; p≤0.0001), and e-cigarette and shisha smokers (mean= 0.53; 95% CI: 0.11–0.95; p=0.007).

**Figure 2:**
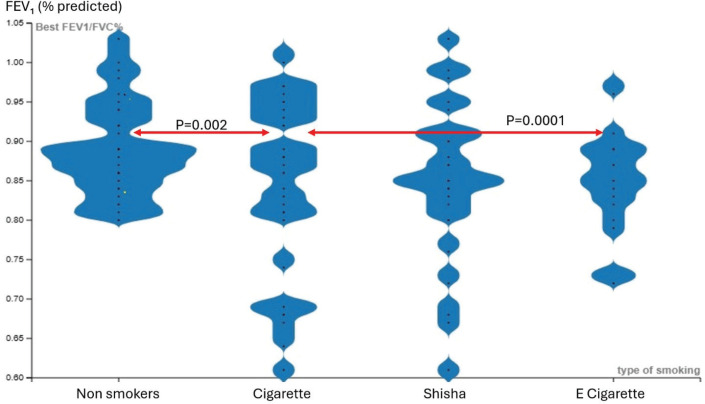
Comparison of the groups best FEV_1_ (% predicted), a cross-sectional observational study conducted in Saudi Arabia, 2023 (N=128)

Regarding FEV_1_ (% predicted), cigarette users had significantly lower values compared to non-smokers and e-cigarette users. A significant difference was observed between at least two groups (p≤0.0001), with *post hoc* analysis identifying a mean difference between non-smokers and cigarette users (mean= 4.74; 95% CI: 3.35–20.68), and a mean difference between e-cigarette and cigarette users (mean=15.01; 95% CI: 5.98–24.05; p=0.0001). Figure 2 shows the comparison of FEV_1_% across the groups.

In terms of forced vital capacity (FVC), cigarette and shisha users had lower values than non-smokers and e-cigarette users. One-way ANOVA showed a significant difference in FVC (p=0.004), with *post hoc* comparisons indicating significant differences between e-cigarette and cigarette users (mean= 0.64; 95% CI: 0.13–1.14; p=0.004). However, the groups had no significant differences in FVC% or FEV_1_/FVC ratio (Table 2).

**Table 2:** Pulmonary function test results, a cross-sectional observational study conducted in Saudi Arabia, 2023 (N=128)

*Parameters*	*Non-smokers* *(N=36)* *Mean ± SD*	*Cigarettes* *(N=30)* *Mean ± SD*	*Shisha* *(N=31)* *Mean ± SD*	*E-cigarettes* *(N=31)* *Mean ± SD*	*p*
FEV_1_ (L/s)	3.72 ± 0.59	3.11 ± 0.54	3.26 ± 0.71	3.79 ± 0.70	≤0.0001
FEV_1_ (% predicted)	93.84 ± 9.38	81.63 ± 12.11	88.09 ± 12.92	96.64 ± 18.62	≤0.0001
FVC (L)	4.26 ± 0.76	3.87 ± 0.68	3.95 ± 0.88	4.51 ± 0.68	0.004
FVC (% predicted)	90.84 ± 8.11	86.10 ± 11.31	91.68 ± 11.05	99.06 ± 16.00	0.145
FEV_1_/FVC (%)	88.61 ± 5.90	83.86 ± 10.95	83.65 ± 10.40	84.93 ± 8.33	0.063

### Hematological and inflammatory parameters

Table 3 presents the hematological and inflammatory data. Cigarette users exhibited significantly higher white blood cell (WBC), neutrophil, lymphocyte, and monocyte counts compared to non-smokers, shisha users, and e-cigarette users. One-way ANOVA tests revealed significant differences for WBC (p≤0.0001), neutrophils (p=0.001), lymphocytes (p≤0.0001), and monocytes (p≤0.0001) among the groups.

**Table 3:** Complete blood count and types of smoking, a cross-sectional observational study conducted in Saudi Arabia, 2023 (N=128)

*Parameters*	*Non-smokers* *(N=36)* *Mean ± SD*	*Cigarettes* *(N=30)* *Mean ± SD*	*Shisha* *(N=31)* *Mean ± SD*	*E-cigarettes* *(N=31)* *Mean ± SD*	*p*
RBC (10^12^/L)	5.16 ± 0.43	5.32 ± 0.57	5.12 ± 0.63	5.20 ± 0.46	0.499
HGB (g/L)	14.95 ± 1.56	18.92 ± 2.23	14.72 ± 1.91	14.63 ± 1.40	0.280
HCT (L/L)	0.46 ± 0.06	0.46 ± 0.06	0.44 ± 0.05	0.44 ± 0.04	0.121
Platelets (10^9^/L)	253.5 ± 61.46	291.5 ± 67.90	266.03 ± 67.98	257.61 ± 71.89	0.109
WBC (10^9^/L)	6.59 ± 1.65	7.92 ± 2.84	5.62 ± 2.12	5.31 ± 2.17	≤0.0001
Eosinophils (10^9^/L)	0.16 ± 0.13	0.19 ± 0.13	0.18 ± 0.17	0.21 ± 0.25	0.778
Neutrophils (10^9^/L)	3.44 ± 1.33	4.03 ± 2.29	2.62 ± 1.30	2.57 ± 1.28	0.001
Lymphocytes (10^9^/L)	2.42 ± 0.61	2.95 ± 0.95	2.19 ± 1.03	2.01 ± 0.82	≤0.0001
Basophils (10^9^/L)	0.034 ± 0.07	0.04 ± 0.08	0.03 ± 0.04	0.03 ± 0.05	0.916
Monocytes (10^9^/L)	0.54 ± 0.13	0.67 ± 0.21	0.47 ± 0.23	0.43 ± 0.24	≤0.0001
CRP (mg/L), median (IQR)	1.0 (1.06)	2.4 (6.66)	2.8 (4.36)	2.2 (4.40)	0.008

RBC: red blood cell count. HGB: hemoglobin. HCT: hematocrit. WBC: white blood cell count. CRP: C-reactive protein.

**Figure 3:**
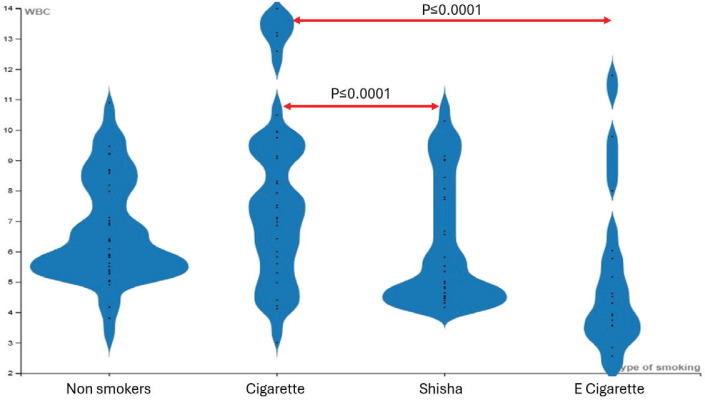
Comparison of white blood cell (WBC) count groups, a cross-sectional observational study conducted in Saudi Arabia, 2023 (N=128)

Further *post hoc* analyses showed cigarette users had significantly higher WBC counts than shisha users (mean=2.3; 95% CI: 0.84–3.75; p≤0.0001) and e-cigarette users (mean=2.61; 95% CI: 1.14–4.08; p≤0.0001) (Figure 3). Neutrophil counts were also significantly higher in cigarette users compared to shisha (mean=1.41; 95% CI: 0.36–2.46; p=0.003) and e-cigarette users (mean=1.46; 95% CI: 0.40–2.52; p=0.003). Higher levels of lymphocyte and monocyte counts were observed among cigarette users, and the difference was statistically significant among the groups. A one-way ANOVA revealed a statistically significant difference between at least two groups (p=0.000).

Tukey’s HSD test for multiple comparisons showed that the mean value lymphocyte count was significantly higher in cigarette smokers compared to e-cigarettes (mean=0.24; 95% CI: 0.367–1.52; p=0.000), and in cigarette smokers compared to shisha smokers (mean=0.20; 95% CI: 0.12–1.34; p=0.003).

**Figure 4:**
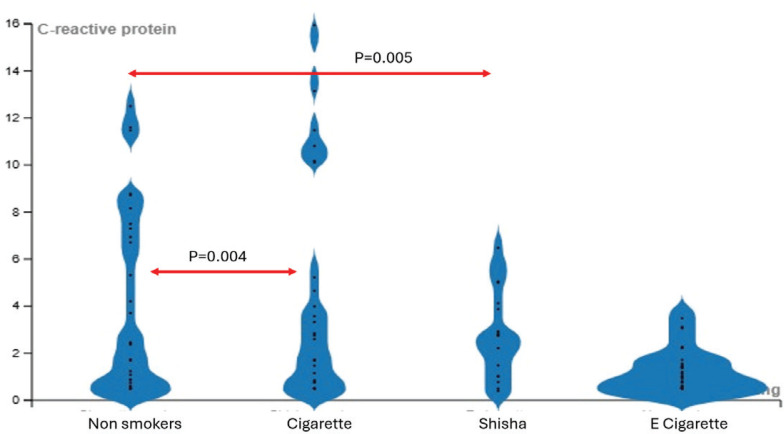
Comparison of C-reactive protein levels across groups, a cross-sectional observational study conducted in Saudi Arabia, 2023 (N=128)

A Kruskal-Wallis H test indicated a significant difference in C-reactive protein (CRP) levels among the groups [H (3)=11.74; p=0.008]. *Post hoc* Dunn’s tests revealed significant differences between the non-smokers group and cigarette smokers (p=0.004), as well as between non-smokers and shisha smokers (p=0.005) (Figure 4).

## DISCUSSION

This study is one of the first comparative analyses of the respiratory, hematological, and inflammatory profiles among long-term users of cigarettes, shisha, and e-cigarettes in Saudi Arabia. Our findings show that cigarette and shisha users exhibit significantly lower FEV_1_ and FEV_1_% compared to non-smokers and e-cigarette users, highlighting the detrimental effects of traditional smoking on lung function. Additionally, both cigarette and shisha users had lower FVC than non-smokers and e-cigarette users, further emphasizing the negative impact of traditional smoking methods on pulmonary health.

Although there was no definitive evidence of airway obstruction, the PFT results among the smoking groups indicate an emerging obstructive pattern. None of the groups had participants with FEV_1_/FVC (%) values below the 70% threshold for obstruction, but borderline values were observed across all groups. This suggests that some individuals may be at risk of early airway limitation. These findings point to subtle, subclinical changes in lung function likely related to smoking, regardless of the method, which could progress with continued exposure. This notable decline in lung function aligns with previous findings that attribute this decline to structural abnormalities such as emphysema and airway narrowing caused by inflammation and mucus accumulation. Shisha smoking, despite the common perception as a safer alternative due to water filtration, also led to significant reductions in lung function. Similar findings have been reported by Meo et al.^23^ and Raad et al.^24^ who observed decreased FEV_1_ and FEV_1_/FVC among shisha smokers.

Interestingly, e-cigarette users exhibited less lung function impairment compared to cigarette and shisha users. However, evidence on the long-term effects of e-cigarettes remains limited. The Polosa et al.^25^ 3.5-year study found no significant changes in lung function parameters among e-cigarette users. Nonetheless, most studies on e-cigarettes have only explored short-term effects, and more longitudinal research is needed to fully assess their long-term impact, especially among dual users of cigarettes and e-cigarettes^26^.

Hematological analyses revealed that cigarette smokers had significantly higher WBC, neutrophil, lymphocyte, and monocyte counts compared to shisha and e-cigarette users, suggesting a heightened inflammatory response. C-reactive protein (CRP) levels were also significantly elevated in cigarette and shisha smokers compared to non-smokers, indicating an increased inflammatory burden associated with traditional smoking. In contrast, e-cigarette users demonstrated relatively better respiratory function and lower inflammatory markers, suggesting a potentially less harmful profile.

The cigarette smoking group exhibited significantly higher hemoglobin levels compared to the other groups. This may be attributed to chronic hypoxemia caused by elevated carboxyhemoglobin levels, which is common in cigarette smokers. Additionally, the longer average smoking duration in the cigarette group (about 7 years) compared to the shisha and e-cigarette groups (about 4 years) likely contributes to this increase in hemoglobin, as prolonged smoking can further stimulate the body to compensate for reduced oxygen levels by increasing hemoglobin production.

Previous studies have reported significant increases in parameters such as hematocrit, hemoglobin, and leukocyte count among tobacco smokers, a trend attributed to carbon monoxide exposure and nicotine’s effects on the body. Our study aligns with these findings, as cigarette users demonstrated significantly higher WBC, neutrophil, lymphocyte, and monocyte counts compared to shisha and e-cigarette users^27-31^. This leukocytosis is likely driven by nicotine-induced catecholamine release and the inflammatory response in the respiratory tract. Furthermore, elevated CRP levels in both cigarette and shisha smokers are consistent with the well-established link between smoking and chronic inflammation^31-34^.

The shisha group had the highest percentage of females (32%), compared to 14% in the cigarette group and 13% in the e-cigarette group. Several factors may explain this trend. Shisha smoking is often perceived as more socially acceptable and less harmful than cigarette or e-cigarette use, particularly in certain communities. It is also commonly enjoyed in group settings such as cafes or social gatherings, which may appeal more to females who prefer these communal experiences. Additionally, shisha smoking is marketed with appealing flavors and aesthetically pleasing environments, further attracting female users. The perception that shisha carries lower health risks due to its filtered smoke may also contribute to its popularity among women. In some cultures, shisha smoking has traditionally been more accepted for females than cigarettes or e-cigarettes, influencing their preferences. The growing popularity of shisha smoking is closely linked to the increasing café culture in the Middle East, noticeable among female gatherings.

While e-cigarette users in our study showed minimal changes in hematological parameters, prior research has indicated that acute e-cigarette use can transiently increase oxidative stress and inflammation, which may pose risks to the vascular system^35,36^. Further long-term studies are necessary to evaluate the potential vascular and systemic effects of e-cigarettes.

### Limitations

This study has several limitations. The cross-sectional design prevents us from establishing causal relationships between smoking types and health outcomes. Self-reported data on smoking duration may also introduce recall bias. Although our sample size was adequate for detecting group differences, it fell short of the calculated target, which may limit the generalizability of our findings. Furthermore, while smoking type and duration were documented, the degree of smoking consumption, including concentration and frequency, was not accounted for, which could influence the observed health impacts. Residual confounding may persist due to unmeasured variables, including lifestyle factors or genetic predispositions, which could have influenced the results. Furthermore, the limited proportion of female participants constrains the generalizability of the findings across genders. Additionally, the study was conducted within a specific geographical and cultural context, so the results may not be fully generalizable to other populations or countries. Future research should adopt a longitudinal design to establish causality and incorporate objective exposure measures, such as biomarkers, to improve accuracy. Additionally, measuring carboxyhemoglobin (Co-Hgb) levels could provide more definitive insights into the relationship between cigarette smoking and elevated hemoglobin levels. It would also be beneficial to account for variations in smoking frequency and intensity when assessing the health impacts of different smoking behaviors.

## CONCLUSIONS

Compared to non-smokers, long-term cigarette and shisha users showed significant changes in pulmonary function, accompanied by abnormally elevated hematological and inflammatory responses. In contrast, e-cigarette users displayed relatively lower inflammatory markers and less deviation from normal lung function, though some exhibited borderline signs of airway obstruction. Further research is essential to fully assess the long-term health effects of e-cigarette use.

## Data Availability

The data supporting this research are available from the authors on reasonable request.
